# The First Wedding

**Published:** 1878-07

**Authors:** 


**The First Wedding.**—We like the short courtships, and in this Adam acted like a sensible man—he fell asleep a bachelor, and awoke to find himself a married man. He appears to have popped the question almost immediately after meeting Miss Eve, and she, without flirtation or shyness, gave him a kiss and herself. Of that first kiss in the world we have had our own thoughts, however, and sometimes, in a poetical mood, wished we were the man that did it. But the deed is done—the chance was Adam's, and he improved it. We like the notion of getting married in a garden. Adam's was private. No envious aunts and grunting grandmothers. The birds of the heavens were the minstrels, and the glad sky flung its light on the scene. One thing about the first wedding brings queer things to us in spite of its scriptural truth. Adam and his wife were rather young to marry; some two or three days old, according to the sagest elder; without experience, without a house, a pot or kettle; nothing but love and Eden.

